# Expansion of effector memory Vδ2^neg^ γδ T cells associates with cytomegalovirus reactivation in allogeneic stem cell transplant recipients

**DOI:** 10.3389/fimmu.2024.1397483

**Published:** 2024-06-10

**Authors:** Yiwen Huang, Cen Jiang, Jiacheng Zhu, Lin Lin, Minjing Mao, Tong Yin, Gang Cai

**Affiliations:** ^1^ Department of Laboratory Medicine, Ruijin Hospital, Shanghai Jiaotong University Medical School, Shanghai, China; ^2^ Shanghai Institute of Hematology, State Key Laboratory of Medical Genomics, National Research Center for Translational Medicine at Shanghai, Ruijin Hospital, Shanghai JiaoTong University School of Medicine, Shanghai, China

**Keywords:** cytomegalovirus, γδ T cells, hematopoietic stem cell transplantation, antiviral immunity, cytotoxicity

## Abstract

**Background:**

Cytomegalovirus (CMV) reactivation is a significant concern following allogeneic stem cell transplantation. While previous research has highlighted the anti-CMV reactivation effect of γδ T cells in immunocompromised transplant patients, their characterization in recipients at high risk of CMV reactivation remains limited.

**Methods:**

This study focused on D+/R+ recipients (where both donor and recipient are CMV seropositive) at high risk of CMV reactivation. We analyzed 28 patients who experienced CMV recurrence within 100 days post-allogeneic hematopoietic stem cell transplantation, along with 36 matched recipients who did not experience CMV recurrence. Clinical data from both groups were compared, and risk factors for CMV reactivation were identified. Additionally, CMV viral load was measured, and flow cytometric analysis was conducted to assess changes in peripheral blood γδ T cell proportions, subpopulation distribution, and differentiation status. We also analyzed the CDR3 repertoire of the TCR δ chain in different γδ T cell subsets. Functional analysis was performed by measuring the lysis of CMV-infected cells upon stimulation.

**Results:**

CMV reactivation post-transplantation was associated with acute graft-versus-host disease (aGvHD) and reactivation of non-CMV herpesviruses. Notably, CMV reactivation led to sustained expansion of γδ T cells, primarily within the Vδ2^neg^ γδ T cell subpopulation, with a trend toward differentiation from Naive to effector memory cells. Analysis of the δ chain CDR3 repertoire revealed a delay in the reconstitution of clonal diversity in Vδ2^neg^ γδ T cells following CMV reactivation, while Vδ2^pos^ T cells remained unaffected. Upon stimulation with CMV-infected MRC5 cells, the Vδ2^neg^ γδ T cell subpopulation emerged as the primary effector cell group producing IFN-γ and capable of lysing CMV-infected cells. Moreover, our findings suggest that NKG2D is not necessary involved in Vδ2^neg^ γδ T cell-mediated anti-CMV cytotoxicity.

**Conclusion:**

This study provides novel insights into the role of γδ T cells in the immune response to CMV reactivation in transplantation recipients at high risk of CMV infection. Specifically, the Vδ2^neg^ γδ T cell subpopulation appears to be closely associated with CMV reactivation, underscoring their potential role in controlling infection and reflecting CMV reactivation in HSCT patients.

## Introduction

Allogeneic hematopoietic stem cell transplantation (allo-HSCT) stands as a pivotal therapeutic intervention saving the lives of numerous patients with hematologic disorders (1). However, post-transplant viral infections emerge as the foremost cause of non-relapse mortality, with Human Cytomegalovirus (CMV) infection leading the charge as the most prevalent (2). While CMV infection typically remains asymptomatic in most healthy individuals, transplant recipients, due to their suppressed immune systems, lack CMV-specific cytotoxic cells and helper T cells, rendering them vulnerable to life-threatening cytomegalovirus disease. Moreover, CMV infection heightens the susceptibility to other pathogenic infections, such as bacteria, fungi, Epstein-Barr virus, varicella-zoster virus, and escalates the incidence of Graft-Versus-Host Disease (GvHD) in patients (3).

The immune response to CMV predominantly hinges upon dendritic cells (DCs), natural killer cells (NK cells), and αβ T lymphocytes (4–7). Recent investigations have illuminated the significant contribution of γδ T cells to T cell-mediated anti-CMV responses (8–11). Comprising the γ and δ chains, the γδ T-cell receptor constitutes approximately 2 to 5% of human peripheral blood lymphocytes, orchestrating immune responses against infections and tumors (12). Often dubbed ‘unconventional’ T cells, γδ T cells, unlike αβ T cells, eschew surface expression of CD4 and CD8 and exhibit non-MHC-restricted recognition (13–15). These cells can be directly activated, proliferate, differentiate into cytotoxic cells, or produce various cytokines in response to a range of antigenic stimuli (16, 17). Within the human body, two major subsets of γδ T cells: Vγ9^+^Vδ2^+^ T cells, expressing a TCR encoded by the Vδ2 and Vγ9 gene segments, predominantly found in peripheral blood, and a minor Vδ1 subset more prevalent at mucosal epithelium sites such as skin and the intestine. An additional small subset of Vδ3^+^ γδ T cells also exists in peripheral blood but represents a minority population comprising less than 0.1% of CD3^+^ T cells (16, 18).

The evidence of γδ T cell anti-CMV activity emerged in kidney transplant patients in 1999 (19). Subsequent research has demonstrated that CMV infection triggers persistent expansion of oligoclonal γδ T cells in the blood of CMV-seropositive individuals, augmenting their cytotoxicity and IFN-γ production to combat the virus (20). In immunocompromised patients post-HSCT, a protective role of Vδ2^neg^ γδ T cells during CMV reactivation was discerned, similar to studies of solid organ allografts (10). However, in regions with high CMV infection prevalence, such as China, allo-HSCT recipients face heightened CMV resurgence risk. Detailed information regarding the γδ T cell response to CMV reactivation in such high-risk recipients remains scarce. Hence, in this study, we scrutinized the relative proportions of Vδ2^neg^ compared with Vδ2^+^ γδ T cells in D+/R+ (both donor and recipient CMV seropositive) allo-HSCT patients with and without CMV reoccurrence. Significant disparities were observed in frequencies, repertoire profiles, and cytotoxic effector function in response to CMV-infected cells between the γδ T-cell subsets. Our findings suggest a protective role of Vδ2^neg^ γδ T cells during CMV reactivation and their potential in reflecting CMV reactivation in immunocompromised HSCT patients.

## Materials and methods

### Study subjects and CMV reactivation monitoring

The study subjects comprised patients who underwent allo-HSCT at the Hematology Center of Ruijin Hospital, affiliated with Shanghai Jiao Tong University School of Medicine. Pre-transplant conditioning regimens, graft-versus-host disease (GvHD) prevention and treatment, as well as infection prevention protocols, were consistent with previous reports (21, 22). Specifically, calcineurin inhibitors with short-term methotrexate and mycophenolate mofetil served as the backbone for the GvHD prophylaxis. Peripheral blood stem cells, mobilized by granulocyte colony-stimulating factor from the donor, constituted the source of hematopoietic stem cell transplants.

All included patients were deemed high-risk for CMV reactivation due to CMV seropositive in both donor and recipients. CMV reactivation was monitored weekly with plasma CMV DNA testing for the initial 3 months post-transplant, bi-weekly during months 4–6, and monthly during months 7–12. In case of DNAemia occurrence, monitoring frequency was adjusted to weekly or bi-weekly. Data were recorded with a maximum follow-up duration of one year. Serum CMV-DNA testing utilized the CMV-DNA quantitative real-time PCR assay (Daan Gene Co., Ltd., Shenzhen), with a cutoff value of 500 IU/mL. Pre-emptive therapy commenced when two consecutive test results exceeded 500 IU/mL or when a single test result surpassed 1000 IU/mL. During the follow-up period, viremia occurrence was defined as CMV reactivation. In total, 28 reactivation cases were included, with a median time from allo-HSCT to CMV reactivation of 41 days (range from 19 to 81 days). Among these 28 patients, six were diagnosed with refractory CMV viremia, in line with previously described diagnostic criteria (23). Thirty-six control cases were selected based on matching criteria including the patient’s primary disease, age, donor-recipient type, pre-transplantation approach. Blood samples were obtained from control patients at time points comparable to the CMV reactivation time points of positive patients. Throughout the study period, all control patients were tested negative for various viruses (CMV, EBV, BKV, JCV, HHV6A/B, VZV), as outlined in **Table 1**.

**Table 1:** Clinical characteristics of recipients with and without CMV reactivation.

Clinical characteristics	CMV reactivation (n=28)	CMV unreactivation (n=36)	*P* value
Median age (range, years)	47 (15-61)	46 (16-69)	0.94
Sex, Male/Female	16/12	19/17	0.80
Median follow-up time (range, days)	284(154-365)	258(136-365)	0.46
Primary diseases, cases (%)			0.87
Acute myeloid leukemia	18 (64.3)	21 (58.3)	
Acute lymphoblastic leukemia	6 (21.4)	11 (30.6)	
Myelodysplastic syndrome	3 (10.7)	3 (8.3)	
Others	1 (3.6)	1 (2.8)	
HCT-CI scores, cases (%)			0.70
0 (Low risk)	23(82.1)	29(80.6)	
1–2(Median risk)	4(14.3)	4(11.1)	
≥ 3(High risk)	1(3.6)	3(8.3)	
Pre-treatment methods, cases (%)			0.19
myeloablative conditioning	25(89.3)	35(97.2)	
Reduced-intensity conditioning	3(10.7)	1(2.8)	
HLA compatibility, cases (%)			0.89
Haploidentical	22(78.6)	28(77.8)	
Matched sibling	2(7.1)	3(8.3)	
Matched unrelated	4(14.2)	7(19.4)	
Blood type differences, cases (%)			0.26
Matched	14(50.0)	20 (55.6)	
Major mismatched	9(32.1)	6(16.7)	
Minor mismatched	3(10.7)	9(25.0)	
Major and minor mismatched	2(7.1)	1(2.8)	
MNC counts in graft, median (range, ×10^8^/kg)	13.1(5.9-22.3)	12.7(4.3-24.1)	0.78
CD34^+^ cell counts in graft, median (range, ×10^6^/kg)	8.4(5.1-15.4)	8.9(2.9-13.9)	0.45
Median time from HSCT to neutrophil engraftment (range)	14(10-24)	13(11-20)	0.66
Median time from HSCT to platelet engraftment (range)	12(10-27)	13(11-25)	0.20
Acute GvHD, cases(%)			
II-IV Grade	15(53.5)	9(25.0)	**0.02**
Chronic GvHD, cases(%)			
Moderate to severe	8(28.5)	7(19.4)	0.39
NCH reactivation, cases (%)	22(78.5)	18(50.0)	**0.02**
EBV	20(71.4)	8(22.2)	**<0.01**
HSV1	6(21.4)	7(19.4)	0.85
HHV6B	7(25.0)	4(11.1)	0.14
VZV	3(10.7)	1(2.8)	0.19
Other virus reactivation, cases (%)			
BKV	8(28.5)	9(25.0)	0.71
JCV	5(17.8)	8(22.2)	0.18

HCT-CI, hematopoietic cell transplantation- specific comorbidity; HLA, human leucocyte antigen; MNC, mononuclear cells; HSCT, hematopoietic stem cell transplantation; GvHD, graft versus host disease; NCH, non-CMV herpesvirus; EBV, Epstein-Barr virus; HSV1, Herpes simplex virus-1; HHV6B, human herpesvirus 6B; VZV, varicella-zoster virus, BKV, BK virus; JCV, John Cunningham virus.

Bold text indicates that these items are statistically different.

### Flow cytometry for peripheral Blood γδ T cells and surface markers

Peripheral blood mononuclear cells (PBMCs) were isolated using density gradient separation with Ficoll (Sigma-Aldrich). Subsequently, 0.5~2.0 × 10^6^ cells were used for analysis. Initially, cells were stained with Fixable Viability Stain-BV510 (FV510, BD Bioscience) at room temperature in the dark for 15 minutes. Following PBS washing, cells were incubated at room temperature in the dark for 20 minutes with appropriate monoclonal antibodies, including APC-anti-CD3 (BD Bioscience), PE-anti-pan TCR γ/δ (BD Bioscience), FITC-anti-TCR Vδ2 (BioLegend), PE/Cy7-anti-CD45RA (BD Bioscience), and PerCP/Cy5.5-anti-CD27 (BD Bioscience). After another washing step, the cells were analyzed using the BD Canto II flow cytometer with Diva software for data collection. FlowJo v10.6.2 software facilitated the analysis of γδ T cell proportions, subset distribution, and surface antigen expression. Data exhibiting γδ T cell proportions of 0% or cell counts < 50 in the γδ T cell gate were excluded from the analysis.

### TCR Vδ 1, 2-chains CDR3 size spectratyping analysis

A previous study indicated that Vδ1 and Vδ3 subsets were the primary components of Vδ2^neg^ γδ T cells, with Vδ1 cells selectively expanded by CMV challenge (9, 24). Peripheral CD4^neg^CD8^neg^CD3^+^ cells were purified using the EasySepTM human T cell isolation kit (Stem cell Technologies) according to the manufacturer’s instructions. Total RNA was extracted from isolated T cell pellets using the RNeasy kit (QIAGEN) and reverse transcribed into complementary DNA (cDNA) following the manufacturer’s protocol (Promega). Each cDNA was subsequently amplified using primers for TCR Vδ-1 chain variable (GCCTTAACCATTTCAGCC), Vδ-2 chain variable (TACCGAGAAAAGGACATCTATGGC), and constant segments (GTCGTGTTGAACTGAACATGTCACTG). Aliquots of 2 μL of the PCR products were further amplified with the same forward primer for each Vδ chain, along with Cδ-FAM-labeled constant primer (5’-ACGGATGGTTTGGTATGAGGCTGA-3’), for 10 cycles under the same PCR conditions. Separation of the labeled products was performed using an ABI 3500 DNA Sequencer (Applied Biosystems). The fluorescence intensity for each sample was then analyzed using GeneScan Version 3.0 software (Applied Biosystems).

### Generation of polyclonal γδ T-cell lines

Fresh PBMCs were isolated from CMV reactivation recipients at +180d post-HSCT. Following the isolation of γδ T cells from PBMC using the TCRγ/δ^+^ T cell isolation kit (Miltenyi Biotec), Vδ2^neg^ γδ T cells were sorted out from γδ T cell population using anti-Vδ2 (BD Biosciences PharMingen) monoclonal antibodies. Sorted cells were then expanded using a modified protocol as previously described (25). Polyclonal γδ T-cell cultures were initiated with 1 μg/mL PHA-L (Sigma-Aldrich), 200 IU/mL of human recombinant IL-2 (R&D Systems), and irradiated allogeneic PBMCs (35 Gy). After 2 to 3 weeks of culture, polyclonal lines were immunophenotyped, and purity (routinely > 95%) was determined through multicolor fluorescent staining.

### CMV infection of MRC5 cells

Human embryonic lung fibroblast MRC5 cells were sourced from the Institute of Cell Biology, Chinese Academy of Sciences. These cells were cultured at 37°C in a 5% CO2 incubator using MEM medium (Gibco) supplemented with 2mM L-glutamine (Sigma-Aldrich) and 10% FCS (Gibco). Fibroblasts utilized were within 32 and 40 passage range and were maintained at 37°C in a humid atmosphere containing 5% CO_2_. The clinical CMV strain TB40/E was obtained from Institute of Virology, Chinese Academy of Sciences. CMV suspensions were generated as previously described (25). Briefly, MRC-5 cells were infected with CMV at a multiplicity of infection (MOI) of 0.1 and incubated for 10 days at 37°C in serum-depleted culture medium. The resulting supernatant was harvested and initially centrifuged for 15 minutes at 1,200 g to eliminate cell debris. Subsequently, the virus was concentrated by centrifugation at 6,900 g for 18 hours. The resulting pellet was resuspended in PBS (1:100) without Ca^2+^ or Mg^2+^ and sonicated in an ultrasonic bath for 2 minutes. The protein concentration of the virus preparation was adjusted to 1 mg/mL, and the material was stored at –70°C. For lysis experiments, MRC5 fibroblasts were incubated with the CMV suspension at a MOI of 1 for 2 h, washed, and then cultured for indicated time at 37°C. Microscopic examination was conducted to confirm infection and assess cytopathic effects. Infected cell layers were washed before being used for coculture experiments.

### Detection of cytokines production

To assess the function of cultured polyclonal T-cells, cultured polyclonal γδ T cells were co-cultured with monolayers of CMV-infected or noninfected MRC5 at a ratio of 5:1 for 6 hours at 37°C. For the blocking assay, polyclonal γδ T cells were preincubated for 1 hour with 20μg/mL of anti-NKG2D (R&D), anti-TCRγ/δ (Beckman), or control mouse IgG. IFN-γ released into the supernatant was quantified by ELISA according to the manufacturer’s recommendations (Bender Medsystems).

To evaluate the responses of freshly isolated γδ T cells to CMV-infected MRC5 cells, peripheral γδ T cells were isolated from PBMC of three recipients without CMV reactivation. Isolated γδ T cells were then co-cultured with monolayers of CMV-infected or noninfected MRC5 at a ratio of 5:1 for 6–8 days at 37°C. During the final 6 hours of co-culture, Brefeldin A (BioLegend) working solution was added at a ratio of 1:1000. After culturing, cells were collected for detection of intracellular IFN-γ in γδ T cells. Surface markers, including CD3, pan TCR γ/δ, and TCR Vδ2, were labeled as described above. Cells were fixed with 1 mL of Cytofix buffer (BioLegend) for 20 minutes, followed by centrifugation to remove the supernatant. Subsequently, cells were washed twice with 2 mL of Cytoperm buffer (BioLegend) and incubated at room temperature, protected from light, with anti-IFN-γ (BioLegend) for 20 minutes. After washing with Cytoperm buffer, cells were collected using a flow cytometer (BD CantoII) for analysis.

### Cytotoxicity assays

To assess the cytotoxic effect of freshly isolated Vδ2neg T cells on CMV-infected cells, Vδ2^neg^ T cells were obtained from three CMV reactivation recipients at day +180 post-transplant. Effector Vδ2^neg^ T cells were co-incubated at effector/target ratios (E:Ts) of 10:1 with CMV-infected MRC5 cells or non-infected MCR5 cells as controls. Cytotoxicity was measured after 4-hour culture at 37°C. For the blocking assay, cultured polyclonal γδ T cells were evaluated for cytotoxicity via flow cytometry after co-culture with monolayer CMV-infected MRC5 cells in the absence or presence of 20 μg/mL of anti-NKG2D, anti-TCRγ/δ or control mouse IgG. Following PBS washing, FV510 was added for cell staining for 15 minutes in PBS. Cells were then stained with PE-anti-pan TCR γ/δ in the dark for an additional 20 minutes. At least 10 000 target cells were acquired after gating out the TCR γ/δ positive cells, and the proportion of FV510-positive cells to TCR γ/δ negative cells were calculated. Background target cell death was determined from cells incubated in the absence of effector cells.

### Statistical analysis

Data processing and statistical graph generation were carried out using IBM SPSS 26.0 and Graphpad Prism 9.5 software. Flow cytometry data were expressed as percentages. Normally distributed quantitative data were described using means (standard deviations) and analyzed for intergroup differences using one-way analysis of variance or independent-sample *t*-tests under conditions of homogeneity of variance. Non-normally distributed quantitative data were analyzed using the Mann-Whitney U test for intergroup differences. Categorical variable data were described using case counts and compared between groups using the chi-square test, with pairwise comparisons performed using Least Significant Difference (LSD) test. A significance level of *P* < 0.05 was considered statistically significant.

## Results

### Subjects’ characteristics

The characteristics of the enrolled patients were summarized in **Table 1**. The median age of the 64 transplant patients was 46 years (range: 15–69 years). Patients were categorized into two groups based on whether CMV reactivation occurred during the follow-up period: the CMV reactivation group (n = 28) and the CMV-negative group (n = 36). No significant differences were observed between the two groups regarding age, gender, hematopoietic cell transplantation-specific comorbidity index (HCT-CI), underlying hematologic diseases, transplant methods, donor sources, or the incidence of chronic GvHD (**Table 1**). However, disparities were noted in the rates of acute GvHD and non-CMV herpesvirus (NCH) reactivation, with the CMV reactivation group experiencing more cases of grade II to IV acute GvHD and NCH reactivation. Specifically, the percentages of EBV reactivation (EBV DNA > 1×10^4^ IU/mL) were 71.4% and 22.2% in the CMV reactivation group and CMV-negative group, respectively (*P* < 0.001, **Table 1**). The reactivation rates of HSV1, HHV6B, and VZV exhibited no significant differences between the two groups (**Table 1**).

### Specific expansion of γδ T cells in HSCT patients with CMV reactivation

**Figure 1:**
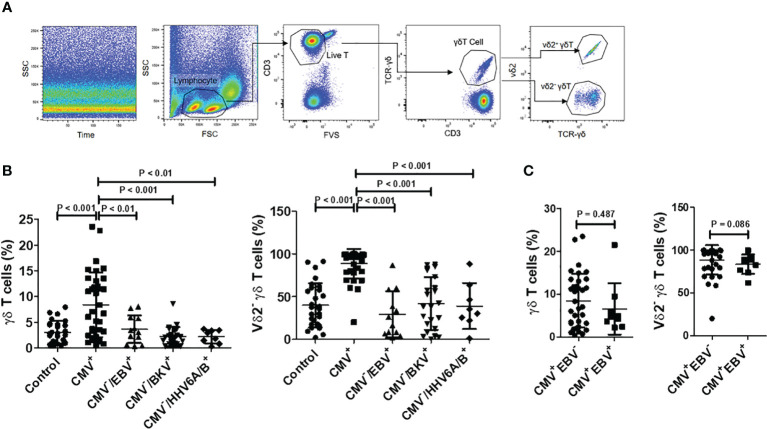
Proportions and subgroup distribution of γδ T cells in CMV reactivation group (CMV^+^) and negative group (CMV^-^). **(A)** Gating strategy: Initially, displacement scattering not produced by laminar flow was excluded based on side scatter (SSC) and Time. Subsequently, lymphocytes were gated based on forward scatter (FSC) and SSC, with a minimum of 5000 lymphocytes counted for percentage determination. Dead cells were excluded based on Fixable Vialbility-BV510 (FV510), and the CD3^+^ T cell population was gated. Within the T cell populations, γδ T cell populations were identified using CD3 and TCRγδ staining. Within the γδ T cell population, Vδ2^+^ γδ T cells and Vδ2^neg^ γδ T cells were gated based on TCRγδ and Vδ2 staining. **(B)** Comparison of γδ T cell levels in total T cells and Vδ2^neg^ γδ T cell levels among control (n = 28), CMV^+^ (n = 36), CMV^-^/EBV^+^ (n = 12), CMV^-^/BKV^+^ (n = 23), and CMV^-^/HHV6A/B^+^ (n = 8) groups. Difference among groups were determined by one-way ANOVA, and Tukey’s test was used for pairwise comparison. **(C)** Comparison of γδ T cell levels in total T cells and Vδ2^neg^ γδ T cell levels between CMV^+^/EBV^-^ (n = 36), CMV^+^/EBV^+^ (n = 9) groups. Mann Whitney test was used to compare these two groups, with *P* < 0.05 considered statistically significant.

To investigate alterations in γδ T cells among HSCT patients in response to CMV reactivation, we initially compared the levels and subset compositions of circulating γδ T cells between CMV-negative patients and those with initial viremia during CMV reactivation. Flow cytometry analysis of γδ T cells and their subsets is depicted in **Figure 1A**. Results revealed that the proportion of γδ T cells to total CD3^+^ T cells in the CMV reactivation group was significantly higher compared to that in the CMV-negative group (8.473% ± 6.240% vs. 3.060% ± 2.118%, *P* < 0.01). Further analysis of the γδ T cell subset composition unveiled that in the CMV-negative group, the majority of γδ T cells expressed Vδ2, with Vδ2^neg^ γδ T cells constituting approximately 39.96% ± 25.75% of total γδ T cells. Conversely, in the CMV reactivation group, there was an inversion in the Vδ2^pos^ to Vδ2^neg^ ratio, with a significantly higher proportion of Vδ2^neg^ γδ T cells, accounting for 88.68% ± 17.14% of total γδ T cells. The difference in the proportion of Vδ2^neg^ γδ T cells to total γδ T cells between the two groups was statistically significant (*P* < 0.001, **Figure 1B**), suggesting an association between CMV reactivation and the expansion of γδ T cells, particularly the Vδ2^neg^ subset.

Analysis of HSCT patients with CMV^neg^/EBV^+^ (n = 12), CMV^neg^/BKV^+^ (n = 23), and CMV^neg^/HHV6A/B^+^ (n = 8) revealed that, in contrast to the negative control group, these patients showed no significant increase in peripheral blood γδ T cell levels and no notable changes in the proportion of Vδ2^+^ to Vδ2^neg^ (**Figure 1B**). Another group of patients with reactivation of both CMV and EBV (CMV^+^/EBV^+^, n = 9) was evaluated, and their levels and constitution of γδ T cell were comparable to those of patients with only CMV reactivation (**Figure 1C**).

### CMV reactivation promotes and maintains expansion of Vδ2^neg^ γδ T cells

**Figure 2:**
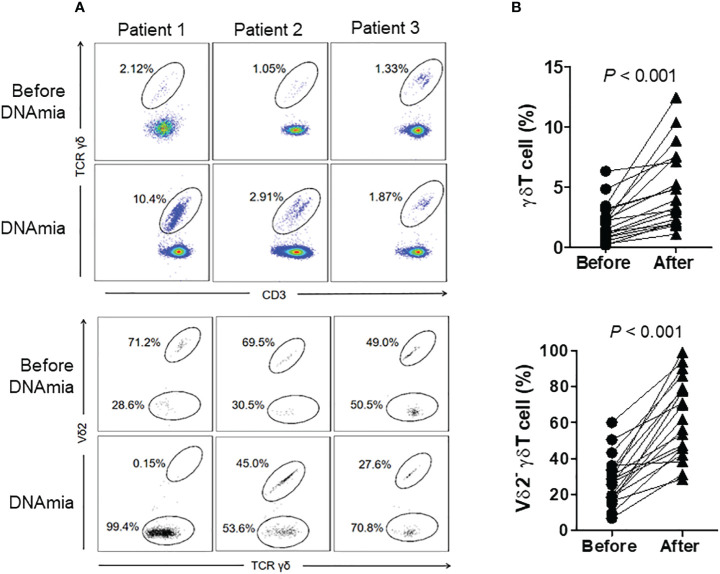
Proportions and distribution of γδ T cell subgroups before and during CMV DNAemia in the same patients. **(A)** Representative flow cytometry results depicting the proportions and distribution of γδ T cell subgroups before and during CMV DNAemia in the same patient. The numbers in the figure indicate the percentages of cells within the gated populations. **(B)** Dynamic changes in the proportions and subgroup distribution of γδ T cells before and after CMV reactivation among 19 patients. Statistical analyses were performed using Mann Whitney test, with a significance level set at 0.05.

To further clarify the association between CMV reactivation and γδ T cells expansion, we compared the levels and subset compositions of γδ T cells before and during DNAemia in HSCT patients (n = 19). The median interval between sampling before DNAemia and DNAemia confirmation was 5 days (range 3~14 days). **Figure 2A** illustrates changes in γδ T cells in three CMV reactivation patients, representative of the nineteen patients, before and at the onset of DNAemia. The proportion of γδ T cells to total CD3^+^ T cells in these patients’ blood increased significantly from 2.10% ± 1.59% before DNAemia to 4.91% ± 3.24% at the time of CMV DNAemia (P < 0.01, **Figure 2B**). Moreover, Vδ2^neg^ γδ T cells expanded significantly, escalating from 27.3% ± 13.6% to 63.0% ± 21.8% of total γδ T cells (P < 0.01, **Figure 2B**).

**Figure 3:**
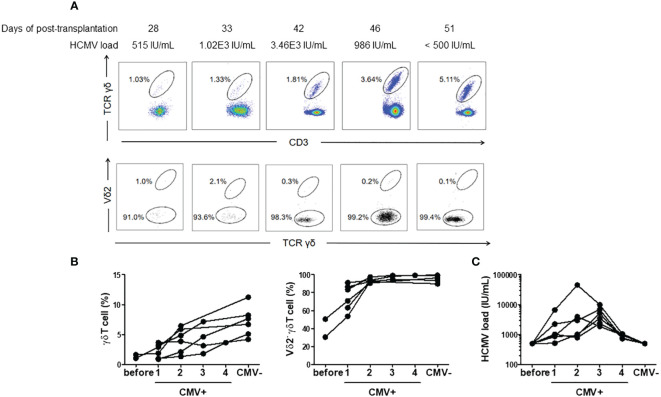
Proportions and subgroup distribution of γδ T cells during CMV reactivation and after clearance of DNAemia. **(A)** Representative flow cytometry results depicting the proportions and subgroup distribution of γδ T cells during CMV reactivation and after clearance of DNAemia. The numbers in the figure represent the percentages of cells within the gated populations. **(B)** Dynamic changes in the proportions and subgroup distribution of γδ T cells before (Before), during CMV reactivation (CMV^+^), and after clearance of DNAemia (CMV^-^) in 6 patients. **(C)** Dynamic changes of CMV load in serum during the analysis of peripheral γδ T cells.

We conducted continuous monitoring from viremia onset to DNAemia clearance in a subset of patients with refractory CMV infection (n = 6). These patients were sampled 4–6 times during the observation period, with a median interval of 6 days between each of the two adjacent analyses (range 3–10 days). The median duration from the last positive sampling and the negative sampling was 7 days (range: 4–12 days). The levels of plasma CMV DNA load compared to γδ T cells and Vδ2^neg^ γδ T cells at different sampling time points in a representative patient are shown in **Figure 3A**. **Figure 3B** demonstrates that γδ T cell levels, particularly Vδ2^neg^ γδ T cells, swiftly increased with CMV DNAemia duration of in these refractory patients. However, even after DNAemia clearance, γδ T cells persisted at a high level, with Vδ2^neg^ cells still predominating. **Figure 3C** shows the corresponding plasma CMV DNA loads at different γδ T cell analysis time points.

### CMV reactivation reshapes γδ T cells differentiation

**Figure 4:**
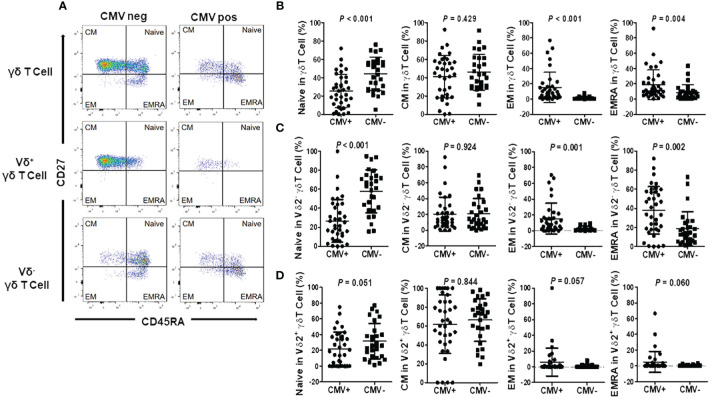
Differentiation of γδ T cells and subgroups in CMV reactivation and negative patients. **(A)** Representative flow cytometry plots illustrating the differentiation of γδ T cells and subgroups in CMV reactivation and negative patients. Cells were categorized into four subgroups based on the differential expression of CD27 and CD45RA: Naive T cells (T _Naive_, CD45RA^+^CD27^+^), Central Memory T cells (T_CM_, CD45RA^-^CD27^+^), Effector Memory T cells (T_EM_, CD45RA^-^CD27^-^), and Effector Memory CD45RA^+^ T cells (T_EMRA_, CD45RA^+^CD27^-^). **(B)** Differentiation status of γδ T cells in CMV reactivation patients (CMV^+^) and negative patients (CMV^-^). **(C)** Differentiation status of Vδ2^+^ γδ T cells in CMV reactivation patients (CMV^+^) and negative patients (CMV^-^). **(D)** Differentiation status of Vδ2^neg^ γδ T cells in CMV reactivation patients (CMV^+^) and negative patients (CMV^-^). Paired t-tests were performed, with *P* < 0.05 considered statistically significant.

To elucidate whether alterations in γδ T cells following CMV reactivation coincide with changes in their differentiation status, we categorized total γδ T cells into distinct subsets based on the expression of CD27 and CD45RA markers (26). These subsets included the Naive (CD45RA^+^CD27^+^), Central Memory (CM, CD45RA^neg^CD27^+^), Effector Memory (EM, CD45RA^neg^CD27^neg^), and Effector Memory CD45RA^+^ cells (EMRA, CD45RA^+^CD27^neg^) as depicted in **Figure 4A**.

Our findings revealed that in the peripheral blood γδ T cell population of CMV reactivation patients, the proportion of Naive cells to total γδ T cells was significantly lower compared to the CMV-negative group, while the proportion of EMRA cells significantly increased. However, there were no statistically significant differences in the proportions of CM and EM cells between the two groups (**Figure 4B**). Further examination of CD27 and CD45RA expression patterns in different γδ T cell subsets, specifically in Vδ2^neg^ γδ T cells and Vδ2^+^ γδ T cells, revealed distinct trends. In the CMV-negative group, Naive cells predominated in the Vδ2^neg^ γδ T cell subset. However, in the CMV reactivation group, this subset exhibited a clear shift toward EM and EMRA phenotype (**Figure 4C**). Conversely, Vδ2^pos^ γδ T cells in the CMV-negative group primarily exhibited CM and Naive phenotypes. In the CMV reactivation group, while a notable increase in EM and EMRA cells was observed in 5 patients, the proportion of EM and EMRA cells did not reach significant difference between the CMV-negative and CMV-reactivation groups (**Figure 4D**). These findings suggest a pivotal role of Vδ2^neg^ γδ T cells in the immune response against CMV infection.

### Expansion of Vδ2^neg^ γδ T cells is clonally restricted

**Figure 5:**
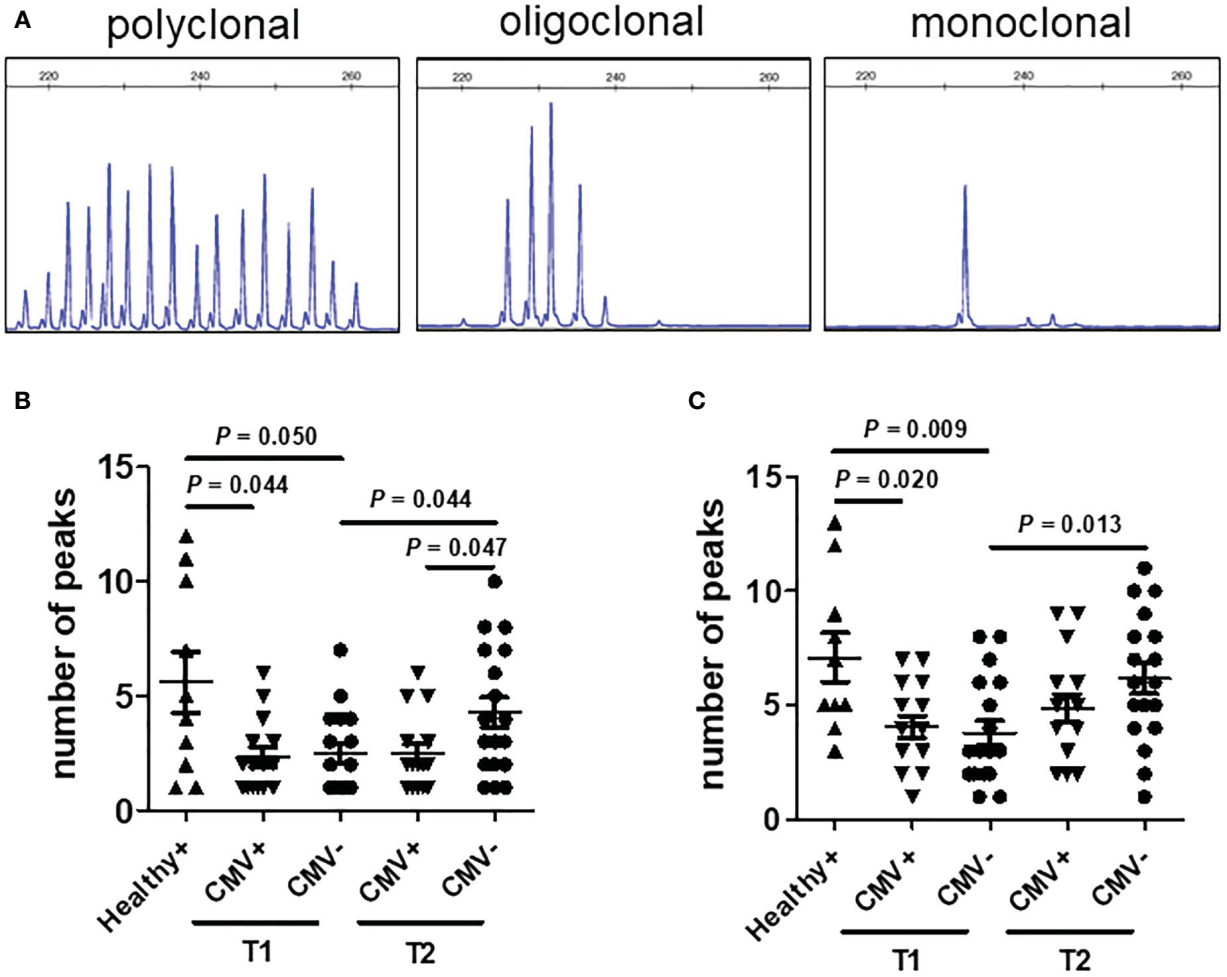
CDR3 spectratyping analysis of the TCR-δ chains in patients after transplantation. **(A)** Representative polyclonal (left), oligoclonal (middle), and monoclonal (right) CDR3 spectratypes of the TCR-δ chain are depicted, expressed as relative fluorescent intensity versus CDR3 length size. Profiles of the CDR3 distribution of Vδ1 **(B)** and Vδ2 **(C)** were generated from PBMCs of healthy seropositive donors (Healthy^+^, n = 10), CMV reactivation recipients (CMV^+^, n = 13) and recipients without CMV reactivation (CMV^-^, n = 18) after allo-HSCT. CMV^+^ recipient samples were collected at the time point of CMV reactivation (T1) and two months later post-DNAemia clearance (T2). CMV^-^ recipient samples were collected at comparable time points as those of CMV^+^ recipients. Samples were analyzed using the nonparametric Mann-Whitney test between indicated groups, and *P <*0.05 considered statistically significant. *P* values more than 0.05 were not shown.

To assess the impact of CMV reactivation on the clonal expansion of proliferated Vδ2^neg^ γδ T cells in patients, we conducted complementary-determining region (CDR3) spectratyping analysis of the TCR-δ1 and TCR-δ2 chain repertoire, as previously reported (10). The complexity of the TCR-δ chain repertoire was evaluated by counting the total number of peaks in each histogram. As a control, analyses were initially performed on seropositive (n=10) and seronegative (n=2) healthy donors. Representative profiles of monoclonal (1 peak), oligoclonal (2–6 peaks), and polyclonal (7 or more peaks) distributions are depicted in **Figure 5A**. Seronegative healthy donors exhibited diverse CDR3 profiles for Vδ1, displaying numerous peaks indicative of various CDR3 length rearrangements. Conversely, seropositive healthy donors showed a range of polyclonal (three cases), oligoclonal, (five cases) and monoclonal (two cases) expansions in Vδ1 cells. Notably, Vδ2 cells displayed comparable polyclonal TCR repertoires in both seropositive and seronegative donors.

In recipients, the reconstituted TCR repertoires of Vδ1 T cells from CMV-reactivated patients (CMV^+^, n = 13) during the CMV activation phase showed similar clonal restriction to those from non-activated patients (CMV^-^, n = 18) at matched post-transplant time points. Furthermore, both CMV^+^ and CMV^-^ recipients exhibited greater clonal restriction in their Vδ1 TCR repertoire compared to healthy donors. Over time, the diversity of TCR repertoires in Vδ1^+^ T cells increased in CMV^-^ patients, but not in CMV^+^ patients. Two months after DNAemia conversion, the CDR3 diversity of Vδ1 T cells was significantly lower in CMV^+^ recipients than in CMV- recipients at the same time after transplantation (**Figure 5B**). Regarding recipient Vδ2^pos^ T cells, the diversity of their CDR3 profiles progressively increased over time after transplantation, regardless of CMV reactivation. There was no significant difference in the diversity of Vδ2^+^ T cell TCR profiles between CMV-positive and CMV-negative patients at matched time points (**Figure 5C**). These results suggest that peripheral expansion of Vδ2^neg^ γδ T cells is clonally restricted during CMV reactivation in patients undergoing allo-HSCT.

### γδ T cells response to CMV-infected MRC5 cells *in vitro*

**Figure 6:**
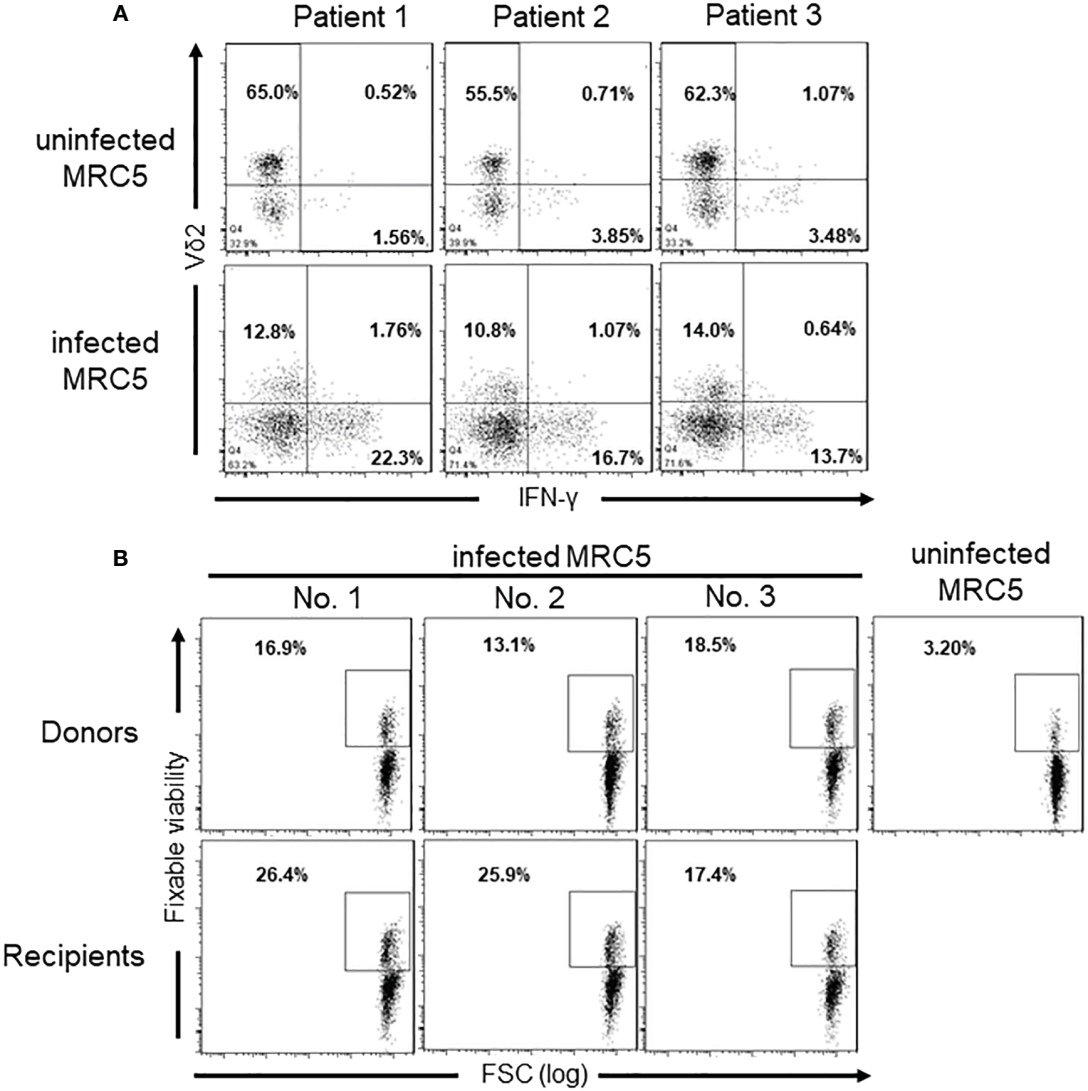
IFN-γ production and cytotoxicity of Vδ2^neg^ γδ T cells against CMV-infected MRC5 cells. **(A)** IFN-γ levels producted by different γδ T cell subgroups after co-culture with CMV-uninfected (CMV^-^) or infected (CMV^+^) MRC5 cells. The ratio of γδ T cells to MRC5 cells is 5:1, and cells cocultured for 6 days. **(B)** Cytotoxicity of Vδ2^neg^ γδ T cells against CMV-infected cells *in vitro*. Monolayers of uninfected or CMV-infected MRC5 fibroblasts were cocultured for 5 hours at 37°C with sorted Vδ2^neg^ γδ T cells from three CMV-seropositive healthy donors and three CMV-reactivation transplantation recipients at the E:T ratio of 10:1. Fixable Viability staining positive cells indicate the lysed MRC5 cells. The numbers in the figure represent the percentages of cells within the gated populations.

To explore the response of γδ T cells to CMV-infected cells *in vitro*, freshly isolated γδ T cells from three HSCT recipients without CMV reactivation were co-cultured with CMV-infected or non-infected MRC5 for several days. The results unveiled that CMV-infected cells significantly spurred the expansion of Vδ2^neg^ γδ T cells and their secretion of IFN-γ in comparison to uninfected cells. Moreover, CMV-infected MRC5 cells notably amplified IFN-γ secretion by Vδ2^neg^ cells, while little such effect was observed within Vδ2^pos^ cells (**Figure 6A**).

Furthermore, cytotoxicity assays revealed that Vδ2^neg^ cells from both seropositive donors and CMV reactivation recipients displayed specific cytotoxicity against CMV-infected fibroblasts, but not against uninfected ones (**Figure 6B**). In contrast, Vδ2^+^ γδ T cells did not exhibit cytotoxicity against CMV-infected cells (**Supplementary Figure 1**). These results unequivocally underscore the anti-CMV capabilities of Vδ2^neg^ T cells, distinguishing them from Vδ2pos T cells.

### Recognition by Vδ2^neg^ γδ T cells of CMV-infected cells is independent of NKG2D and NKG2C ligands

**Figure 7:**
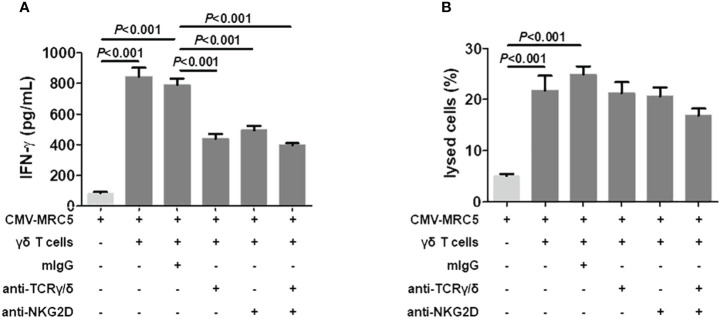
NKG2D-independent recognition of CMV-infected cells by Vδ2^neg^ γδ T cells. **(A)** Detection of IFN-γ secretion in the culture supernatants after 6-hour stimulation of Vδ2^neg^ γδ T cell lines raised from CMV-reactivation recipients (n = 5) with CMV-infected fibroblasts at an E:T ratio of 10:1 in the presence of mouse IgG (mIgG), anti-NKG2D, anti-TCR-γ/δ, and anti-TCR-γ/δ + anti-NKG2D. Supernatant from CMV-infected MRC5 cells was included as control. **(B)** Coculture of CMV-infected MRC5 fibroblasts with Vδ2^neg^ γδ T polyclonal cell lines in the presence of mouse IgG (mIgG), anti-NKG2D, anti-TCR-γ/δ, and anti-TCR-γ/δ + anti-NKG2D. After 6 hours of culture, the percentage of lysed cells among total MRC5 cells was quantified. One-way ANOVA and Tukey’s test were used for statistical analysis, with *P* < 0.05 considered statistically significant.

To clarify the involvement of NKG2D and NKG2C in Vδ2^neg^ γδ T cells’ response to CMV-infected MRC5 cells, Vδ2^neg^ γδ T cells from 5 CMV reactivation recipients were evaluated the expression of NKG2D and NKG2C. In accordance with previous reports (9, 25), NKG2D demonstrated consistent surface expression on the surface of all five cases (**Supplementary Figure 2**). In contrast, NKG2C is barely expressed in Vδ2^neg^ cells. These findings suggest that NKG2C might not play a significant role in the recognition of CMV-infected cells by γδ T cells. Further investigation into the role of NKG2D in cell lysis involved co-culturing Vδ2^neg^ γδ T-cell lines with CMV-infected fibroblasts in the presence or absence of blocking anti-TCRγ/δ and anti-NKG2D antibodies. Surprisingly, blocking TCRγ/δ and NKG2D, either separately or in combination, led to a decrease in IFN-γ production by T cells (**Figure 7A**). However, the specific lysis of CMV-infected targets by cultured γδ T cells remained unaffected by blocking antibodies (**Figure 7B**).

## Discussion

In addition to αβ T cells and NK cells, the distinctive immune properties of γδ T cells position them as another crucial subpopulation in immunocompromised settings for combatting CMV infection. Firstly, γδ T cells directly recognize viruses or virus-induced antigens in an MHC-independent manner (27), offering a unique advantage against viruses like CMV that inhibit MHC antigen expression (28). Secondly, common immunosuppressive drugs such as cyclosporine A are often administered to transplant patients, and γδ T cells have been shown to counteract the effects of such drugs *in vivo* (29). Thirdly, γδ T cells predominantly reside in mucosal epithelial tissues, precisely where CMV replication occurs (30). These factors underscore the growing significance of γδ T cells in the context of combating CMV infection.

In China, the high prevalence of CMV infection means that the majority of Chinese transplant recipients are D^+^/R^+^ patients at high risk of CMV recurrence. However, little is known about the pattern of γδ T cell changes and their response to CMV recurrence in such recipients. In this study, we observed significant expansion of γδ T cells in the peripheral blood of D^+^/R^+^ recipients after CMV reactivation. Additionally, the dominance of γδ T cell subtypes shifted from Vδ2^pos^ to Vδ2^neg^ subgroups. Notably, recipients without CMV reactivation did not exhibit significant expansion or subtype conversion of γδ T cells, suggesting a specific response of γδ T cells to CMV reactivation. This finding aligns with previous observations in renal transplant patients (31) and *in vitro* γδ T cell cytotoxicity experiments (25). The preferential expansion of Vδ2^neg^ cells may be attributed to their antigen recognition properties. Unlike Vδ2^+^ cells, which primarily recognize non-peptidic phosphorylated antigens (32), Vδ2^neg^ cells predominantly recognize stress-induced antigens expressed by epithelial cells, lymphoma cells, and other inducible sources, with CMV infection being a significant stress inducer for epithelial cells (33). Serial monitoring of patients with refractory CMV infection showed that expansion of Vδ2^neg^ γδ T cells occurred early at the onset of viremia, peaked rapidly, and remained at high levels in peripheral blood after viremia turned negative, indicating a potential memory expansion of Vδ2^neg^ γδ T cells following antigenic stimulation (9, 34, 35). Such memory expansion has been implicated in the successful control of latent CMV infection in the elderly (36).

We further analyzed the differentiation status of γδ T cells in HSCT patients. The results indicated that CMV reactivation led to a shift in γδ T cells from Naive to EMRA subsets, as evidenced by the loss of the CD27 antigen. In patients without CMV reactivation, the Vδ2^neg^ subgroup was mainly composed of Naive cells, whereas in CMV reactivation recipients, there was a significant expansion of the EMRA subgroup. T_EMRA_ cells have previously been shown to possess cytotoxic activity against latent virus infections and exhibit highly migratory capabilities with potent effector functions (13). Therefore, the change in the differentiation state of γδ T cells, particularly the Vδ2^neg^ sub-population, upon CMV reactivation likely reflects their attempt to generate a positive immune response to the virus and clear the infection.

It has been reported that Vδ2^+^ cells exhibit a TCR repertoire characterized by stable diversity over the course of an individual’s life, whereas that of Vδ2^neg^ cells tends to decrease during adulthood, which is associated with CMV infection (24). To analyze the impact of CMV reactivation on the repertoire of the γδ T cells, we employed TCR-δ chain CDR3 spectratyping for the TCR-δ chains Vδ1 and Vδ2 subsets in patients after transplantation and in healthy donors. The results showed that the Vδ1 cells were susceptible to CMV infection in both seropositive healthy donors and transplant recipients. Patients without DNAemia after transplantation showed a gradual increase in the TCR diversity of their Vδ1 cells over time, but the recovery of TCR profiles of Vδ1 cells in patients with recurrence of CMV was markedly impeded. This result is similar to what has recently been found in patients with common variable immunodeficiency infected with CMV (37). Unlike Vδ1 cells, the TCR profiles of Vδ2 cells tended to increase progressively in transplant recipients regardless of CMV reactivation. These results suggest that the γδ T cells that undergo reactive clonal expansion upon CMV infection or reactivation primarily belong to the Vδ2^neg^ subset. It is essential to acknowledge that Vδ2^neg^ γδ T cells are not the sole cells capable of responding to CMV infection. CMV reactivation also leads to a significant expansion of Some Vδ2^+^ γδ T cell clones (31, 38), such as the Vγ9^neg^Vδ2^+^ T cells observed in kidney transplant patients in response to CMV reactivation, with this expansion correlating with the severity of CMV infection (31). Consequently, future studies employing single-cell sequencing and other analytical techniques (38) may elucidate the oligoclonal characteristics of γδ T cells that respond to CMV and facilitate the identification of potential CMV-associated antigens.

To address whether expanded Vδ2^neg^ γδ T cells were CMV-reactive, we first identified Vδ2^neg^ cells as the main IFN-γ-producing effector cells by co-culturing T cells derived from CMV-unreactivated transplant recipients with CMV-infected MRC5 fibroblasts. Subsequent cell lysis experiments further confirmed that Vδ2^neg^ cells, either from healthy donors or CMV-reactivated recipients, effective lysed virus-infected fibroblasts, whereas Vδ2^+^ cells did not. These results further emphasize the critical role of Vδ2^neg^ cells in countering CMV reactivation in HSCT recipients.

Previous studies have described that Vδ1 γδ T cells recognized stress-induced antigens, such as MHC class I-related chain A (MICA and MICB), expressed on epithelial tumor cells through the ligand, natural killer group member 2-D (NKG2D) (39). However, whether NKG2D is involved in Vδ2^neg^ γδ T cells recognizing CMV-infected cells remains contentious (10, 11, 25). NKG2C has also been demonstrated to respond to acute CMV infection in recipients of HSCT (40), and is induced on γδ T cells from infants with primary CMV infection (41). In our study, initial analysis of NKG2D and NKG2C expression levels excluded the possibility of NKG2C involvement in the anti-CMV response of γδ T cells, as NKG2C was rarely expressed on Vδ2^neg^ γδ T cells. Furthermore, it appears that NKG2D is not necessary for Vδ2^neg^ γδ T-cell-mediated anti-CMV cytotoxicity. This is supported by the observation that although blocking NKG2D reduced IFN-γ production by cultured γδ T cells, it did not affect the lysis of CMV-infected cells. These findings suggest that while both NKG2D and NKG2C have demonstrated responsiveness to CMV infection and recognition of stress-induced antigens (25, 39–41), the lysis of CMV-infected cells by Vδ2^neg^ γδ T cells may rely more on other co-receptors. Further studies are needed to address this issue.

In conclusion, this study delineates the role of γδ T cells in the immune response against CMV reactivation in allo-HSCT transplant recipients, particularly high-risk D^+^/R^+^ patients. The implications of this study are profound. Firstly, the results showcase the antiviral capacity of Vδ2^neg^ γδ T cells directed toward CMV-infected cells, potentially opening avenues for novel immunotherapy in allogeneic transplantation recipients. Additionally, this study underscores the importance of preserving γδ T cells in the grafts to maintain resistance to CMV infection. Finally, quantification of Vδ2^neg^ γδ T cells in blood proves beneficial for detecting anti-CMV immune responses in transplant recipients. Current diagnostic methodologies for detecting CMV-specific T cells typically involve restimulating CD4^+^ and/or CD8^+^ effector cells with overlapping peptide pools, pre-selected CMV immunodominant peptide cocktails, or CMV-infected cell lysates. Subsequently, induced cytokine production (e.g. IFN-γ) or cell proliferation, is measured using techniques such as flow cytometry, enzyme-linked immunosorbent assay (ELISA) or enzyme-linked immunospot assay (ELISpot) (42, 43). These methods are often labor-intensive and time-consuming, with limited standardization. A novel approach has recently emerged, involving direct staining with CMV polypeptide polymers and enumerate CMV-specific CD8^+^ T cells by flow cytometry (44). However, this method lacks functional readouts and is restricted to certain HLA types, limiting its utility in routine diagnosis. In contrast, peripheral blood Vδ2^neg^ γδ T-cell assays offer a one-step direct staining approach in whole blood using anti-CD3, anti-pan δ, and anti-Vδ2 antibodies, potentially offering a more cost-effective and convenient method if its reliability can match that of the CMV-specific αβ T cell assay.

## Data availability statement

The original contributions presented in the study are included in the article/**Supplementary Materials**, further inquiries can be directed to the corresponding author/s.

## Ethics statement

The studies involving humans were approved by ethics committee of Ruijin Hospital, Shanghai Jiaotong University Medical School. The studies were conducted in accordance with the local legislation and institutional requirements. Written informed consent for participation in this study was provided by the participants’ legal guardians/next of kin.

## Author contributions

YH: Data curation, Formal analysis, Investigation, Methodology, Writing – original draft. CJ: Data curation, Formal analysis, Investigation, Methodology, Writing – original draft, Writing – review & editing. JZ: Formal analysis, Investigation, Methodology, Writing – original draft. LL: Conceptualization, Data curation, Investigation, Software, Writing – review & editing. MM: Data curation, Formal analysis, Investigation, Methodology, Writing – original draft. TY: Conceptualization, Data curation, Formal analysis, Investigation, Writing – review & editing. GC: Conceptualization, Data curation, Formal analysis, Methodology, Supervision, Writing – original draft, Writing – review & editing.

## References

[1] Ljungman P de la Camara R Robin C Crocchiolo R Einsele H Hill JA. Guidelines for the management of cytomegalovirus infection in patients with haematological Malignancies and after stem cell transplantation from the 2017 European Conference on Infections in Leukaemia (ECIL 7). Lancet Infect Dis (2019) 19:e260–e72. doi: 10.1016/S1473-3099(19)30107-0 31153807

[2] Borchers S Luther S Lips U Hahn N Kontsendorn J Stadler M. Tetramer monitoring to assess risk factors for recurrent cytomegalovirus reactivation and reconstitution of antiviral immunity post allogeneic hematopoietic stem cell transplantation. Transpl Infect Dis (2011) 13:222–36. doi: 10.1111/j.1399-3062.2011.00626.x 21585633

[3] Kimura SI Tamaki M Okinaka K Seo S Uchida N Igarashi A. Cytomegalovirus reactivation is associated with an increased risk of late-onset invasive aspergillosis independently of grade II-IV acute graft-versus-host disease in allogeneic hematopoietic stem cell transplantation: JSTCT Transplant Complications Working Group. Ann Hematol (2021) 100:3029–38. doi: 10.1007/s00277-021-04660-3 34490500

[4] La Rosa C Diamond DJ. The immune response to human CMV. Future Virol (2012) 7:279–93. doi: 10.2217/fvl.12.8 PMC3539762 23308079

[5] Gamadia LE Remmerswaal EB Weel JF Bemelman F van Lier RA Ten Berge IJ. Primary immune responses to human CMV: a critical role for IFN-gamma-producing CD4+ T cells in protection against CMV disease. Blood (2003) 101:2686–92. doi: 10.1182/blood-2002-08-2502 12411292

[6] Gillespie GM Wills MR Appay V O'Callaghan C Murphy M Smith N. Functional heterogeneity and high frequencies of cytomegalovirus-specific CD8(+) T lymphocytes in healthy seropositive donors. J Virol (2000) 74:8140–50. doi: 10.1128/JVI.74.17.8140-8150.2000 PMC112348 10933725

[7] Guma M Angulo A Vilches C Gomez-Lozano N Malats N Lopez-Botet M. Imprint of human cytomegalovirus infection on the NK cell receptor repertoire. Blood (2004) 104:3664–71. doi: 10.1182/blood-2004-05-2058 15304389

[8] Prinz I Koenecke C. Antigen-specific gammadelta T cells contribute to cytomegalovirus control after stem cell transplantation. Curr Opin Immunol (2023) 82:102303. doi: 10.1016/j.coi.2023.102303 36947903

[9] Pitard V Roumanes D Lafarge X Couzi L Garrigue I Lafon ME. Long-term expansion of effector/memory Vdelta2-gammadelta T cells is a specific blood signature of CMV infection. Blood (2008) 112:1317–24. doi: 10.1182/blood-2008-01-136713 PMC2515135 18539896

[10] Knight A Madrigal AJ Grace S Sivakumaran J Kottaridis P Mackinnon S. The role of Vdelta2-negative gammadelta T cells during cytomegalovirus reactivation in recipients of allogeneic stem cell transplantation. Blood (2010) 116:2164–72. doi: 10.1182/blood-2010-01-255166 20576814

[11] Liu R Wu N Gao H Liang S Yue K Dong T. Distinct activities of Vdelta1(+) T-cells upon different cytomegalovirus reactivation status after haematopoietic transplantation. Immunology (2022) 167:368–83. doi: 10.1111/imm.13542 35795896

[12] McGuire HM Rizzetto S Withers BP Clancy LE Avdic S Stern L. Mass cytometry reveals immune signatures associated with cytomegalovirus (CMV) control in recipients of allogeneic haemopoietic stem cell transplant and CMV-specific T cells. Clin Transl Immunol (2020) 9:e1149. doi: 10.1002/cti2.1149 PMC7332355 32642063

[13] Ribot JC Lopes N Silva-Santos B. gammadelta T cells in tissue physiology and surveillance. Nat Rev Immunol (2021) 21:221–32. doi: 10.1038/s41577-020-00452-4 33057185

[14] Constantinides MG Belkaid Y. Early-life imprinting of unconventional T cells and tissue homeostasis. Science (2021) 374:eabf0095. doi: 10.1126/science.abf0095 34882451 PMC8697520

[15] Hu W Shang R Yang J Chen C Liu Z Liang G. Skin gammadelta T cells and their function in wound healing. Front Immunol (2022) 13:875076. doi: 10.3389/fimmu.2022.875076 35479079 PMC9035842

[16] Hayday AC. [gamma][delta] cells: a right time and a right place for a conserved third way of protection. Annu Rev Immunol (2000) 18:975–1026. doi: 10.1146/annurev.immunol.18.1.975 10837080

[17] Carding SR Egan PJ. Gammadelta T cells: functional plasticity and heterogeneity. Nat Rev Immunol (2002) 2:336–45. doi: 10.1038/nri797 12033739

[18] Xiong N Raulet DH. Development and selection of gammadelta T cells. Immunol Rev (2007) 215:15–31. doi: 10.1111/j.1600-065X.2006.00478.x 17291276

[19] Dechanet J Merville P Lim A Retiere C Pitard V Lafarge X. Implication of gammadelta T cells in the human immune response to cytomegalovirus. J Clin Invest (1999) 103:1437–49. doi: 10.1172/JCI5409 PMC408467 10330426

[20] Khairallah C Dechanet-Merville J Capone M. gammadelta T cell-mediated immunity to cytomegalovirus infection. Front Immunol (2017) 8:105. doi: 10.3389/fimmu.2017.00105 28232834 PMC5298998

[21] Jiang JL Gao WH Wang LN Wan M Wang L Hu J. Low incidence of relapse with a moderate conditioning regimen of fludarabine, busulfan, and melphalan for patients with myeloid Malignancies: A single-center analysis of 100 patients. Transplant Cell Ther (2023) 29:512 e1–e8. doi: 10.1016/j.jtct.2023.05.017 37263418

[22] Zhang Y Zhang Y Chen Q Tang G Zhang W Yang J. Allogeneic hematopoietic stem cells transplantation improves the survival of intermediate-risk acute myeloid leukemia patients aged less than 60 years. Ann Hematol (2019) 98:997–1007. doi: 10.1007/s00277-018-3584-2 30607578

[23] Stem Cell Application Group CSoHCMA. [The Chinese consensus on the management of cytomegalovirus infection in allogeneic hematopoietic stem cell transplantation patients (2022)]. Zhonghua Xue Ye Xue Za Zhi (2022) 43:617–23. doi: 10.3760/cma.j.issn.0253-2727.2022.08.001 PMC9593016 36709144

[24] Davey MS Willcox CR Joyce SP Ladell K Kasatskaya SA McLaren JE. Clonal selection in the human Vdelta1 T cell repertoire indicates gammadelta TCR-dependent adaptive immune surveillance. Nat Commun (2017) 8:14760. doi: 10.1038/ncomms14760 28248310 PMC5337994

[25] Halary F Pitard V Dlubek D Krzysiek R de la Salle H Merville P. Shared reactivity of Vdelta2(neg) gammadelta T cells against cytomegalovirus-infected cells and tumor intestinal epithelial cells. J Exp Med (2005) 201:1567–78. doi: 10.1084/jem.20041851 PMC2212929 15897274

[26] Mahnke YD Brodie TM Sallusto F Roederer M Lugli E. The who's who of T-cell differentiation: human memory T-cell subsets. Eur J Immunol (2013) 43:2797–809. doi: 10.1002/eji.201343751 24258910

[27] Sciammas R Kodukula P Tang Q Hendricks RL Bluestone JA. T cell receptor-gamma/delta cells protect mice from herpes simplex virus type 1-induced lethal encephalitis. J Exp Med (1997) 185:1969–75. doi: 10.1084/jem.185.11.1969 PMC2196341 9166426

[28] Tomazin R Boname J Hegde NR Lewinsohn DM Altschuler Y Jones TR. Cytomegalovirus US2 destroys two components of the MHC class II pathway, preventing recognition by CD4+ T cells. Nat Med (1999) 5:1039–43. doi: 10.1038/12478 10470081

[29] Lin T Matsuzaki G Umesue M Omoto K Yoshida H Harada M. Development of TCR-gamma delta CD4-CD8+ alpha alpha but not TCR-alpha beta CD4-CD8+ alpha alpha i-IEL is resistant to cyclosporin A. J Immunol (1995) 155:4224–30. doi: 10.4049/jimmunol.155.9.4224 7594578

[30] Boismenu R Havran WL. Modulation of epithelial cell growth by intraepithelial gamma delta T cells. Science (1994) 266:1253–5. doi: 10.1126/science.7973709 7973709

[31] Kaminski H Menard C El Hayani B Adjibabi AN Marseres G Courant M. Characterization of a unique gammadelta T-cell subset as a specific marker of cytomegalovirus infection severity. J Infect Dis (2021) 223:655–66. doi: 10.1093/infdis/jiaa400 32622351

[32] Das H Wang L Kamath A Bukowski JF. Vgamma2Vdelta2 T-cell receptor-mediated recognition of aminobisphosphonates. Blood (2001) 98:1616–8. doi: 10.1182/blood.V98.5.1616 11520816

[33] Eberl M Jomaa H Hayday AC. Integrated immune responses to infection - cross-talk between human gammadelta T cells and dendritic cells. Immunology (2004) 112:364–8. doi: 10.1111/j.1365-2567.2004.01921.x PMC1782494 15196203

[34] Alejenef A Pachnio A Halawi M Christmas SE Moss PA Khan N. Cytomegalovirus drives Vdelta2neg gammadelta T cell inflation in many healthy virus carriers with increasing age. Clin Exp Immunol (2014) 176:418–28. doi: 10.1111/cei.12297 PMC4008987 24547915

[35] Roux A Mourin G Larsen M Fastenackels S Urrutia A Gorochov G. Differential impact of age and cytomegalovirus infection on the gammadelta T cell compartment. J Immunol (2013) 191:1300–6. doi: 10.4049/jimmunol.1202940 23817410

[36] Thome JJ Yudanin N Ohmura Y Kubota M Grinshpun B Sathaliyawala T. Spatial map of human T cell compartmentalization and maintenance over decades of life. Cell (2014) 159:814–28. doi: 10.1016/j.cell.2014.10.026 PMC4243051 25417158

[37] Chan S Morgan B Yong MK Margetts M Farchione AJ Lucas EC. Cytomegalovirus drives Vdelta1(+) gammadelta T cell expansion and clonality in common variable immunodeficiency. Nat Commun (2024) 15:4286. doi: 10.1038/s41467-024-48527-3 38769332 PMC11106253

[38] Ravens S Schultze-Florey C Raha S Sandrock I Drenker M Oberdorfer L. Human gammadelta T cells are quickly reconstituted after stem-cell transplantation and show adaptive clonal expansion in response to viral infection. Nat Immunol (2017) 18:393–401. doi: 10.1038/ni.3686 28218745

[39] Groh V Rhinehart R Secrist H Bauer S Grabstein KH Spies T. Broad tumor-associated expression and recognition by tumor-derived gamma delta T cells of MICA and MICB. Proc Natl Acad Sci U S A (1999) 96:6879–84. doi: 10.1073/pnas.96.12.6879 PMC22010 10359807

[40] Ishiyama K Arakawa-Hoyt J Aguilar OA Damm I Towfighi P Sigdel T. Mass cytometry reveals single-cell kinetics of cytotoxic lymphocyte evolution in CMV-infected renal transplant patients. Proc Natl Acad Sci U.S.A (2022) 119(8):e2116588119. doi: 10.1073/pnas.2116588119 35181606 PMC8872722

[41] Tuengel J Ranchal S Maslova A Aulakh G Papadopoulou M Drissler S. Characterization of Adaptive-like gammadelta T Cells in Ugandan Infants during Primary Cytomegalovirus Infection. Viruses (2021) 13(10):1987. doi: 10.3390/v13101987 34696417 PMC8537190

[42] Maecker HT Maino VC. Analyzing T-cell responses to cytomegalovirus by cytokine flow cytometry. Hum Immunol (2004) 65:493–9. doi: 10.1016/j.humimm.2004.02.004 15172449

[43] Abu-Khader A Krause S. Rapid monitoring of immune reconstitution after allogeneic stem cell transplantation–a comparison of different assays for the detection of cytomegalovirus-specific T cells. Eur J Haematol (2013) 91:534–45. doi: 10.1111/ejh.12187 23952609

[44] Morita-Hoshi Y Heike Y Kawakami M Sugita T Miura O Kim SW. Functional analysis of cytomegalovirus-specific T lymphocytes compared to tetramer assay in patients undergoing hematopoietic stem cell transplantation. Bone Marrow Transplant (2008) 41:515–21. doi: 10.1038/sj.bmt.1705932 18026143

