# Supplementary figures and images for: Expansion of effector memory Vδ2neg γδ T cells associates with cytomegalovirus reactivation in allogeneic stem cell transplant recipients
Source: Front Immunol. 2024 Jun 10;15:1397483. doi: 10.3389/fimmu.2024.1397483 (PMC11194311; doi:10.3389/fimmu.2024.1397483)

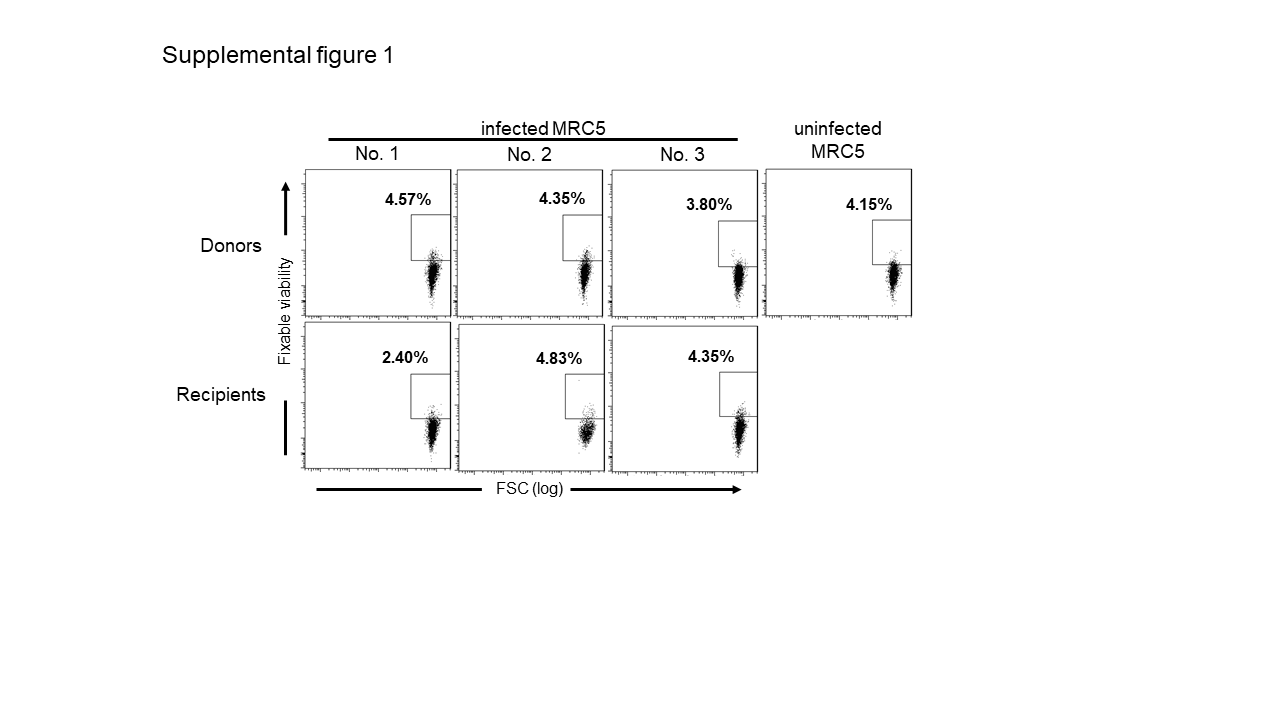

Supplement: Supplementary file 1 [file Image_1.tif]

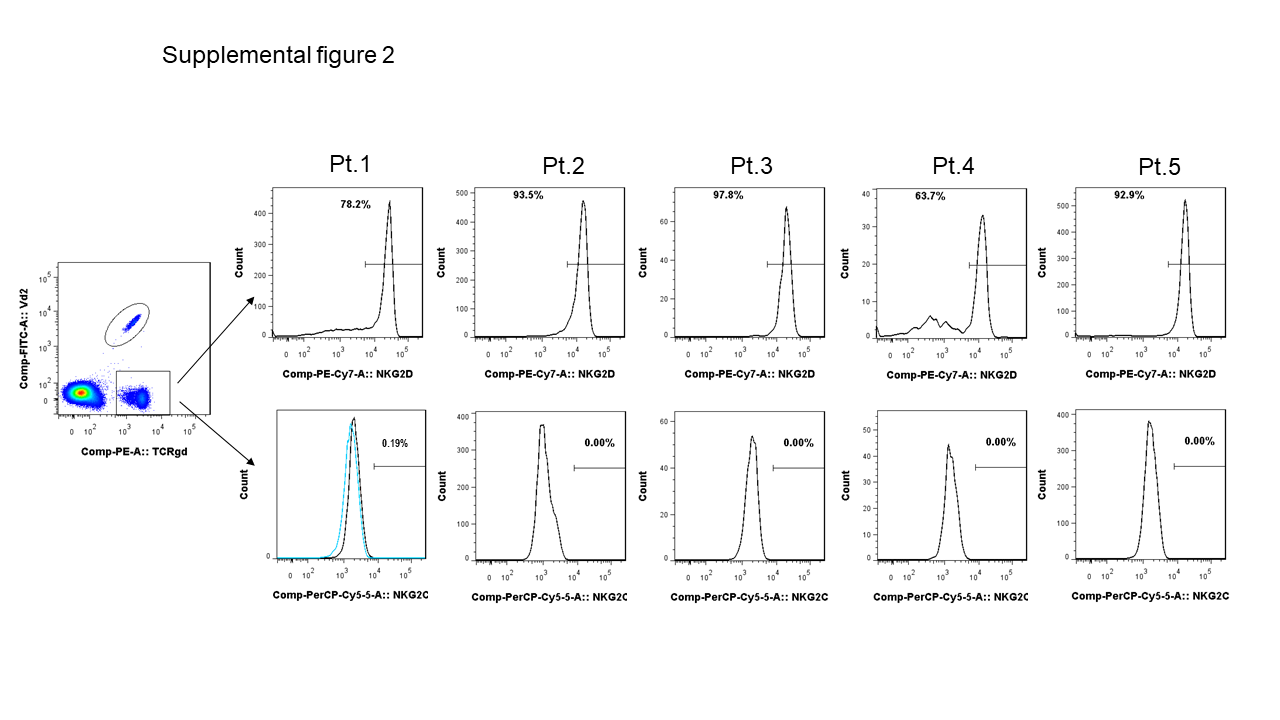

Supplement: Supplementary file 2 [file Image_2.tif]
